# Comparison of Intra-articular Knee Injection of Corticosteroid between Hemodialysis and Non-hemodialysis Patients

**DOI:** 10.31662/jmaj.2023-0020

**Published:** 2023-06-12

**Authors:** Yusuke Tabata, Satoshi Omori, Osamu Mitsuhashi, Kazuo Enomoto, Yuki Sawano, Noriyoshi Murotani, Koichiro Omori, Yoichiro Tabata, Tokifumi Majima

**Affiliations:** 1 Department of Orthopedic Surgery, Mitsuhashi Hospital, Chiba, Japan; 2 Vascular Access Center, Mitsuhashi Hospital, Chiba, Japan; 3 Department of Orthopedic Surgery, Nippon Medical School Hospital, Tokyo, Japan

**Keywords:** Hemodialysis, Steroid injection, Knee osteoarthritis, Complication

## Abstract

**Introduction:**

Hemodialysis patients have various complications, and orthopedic diseases include carpal tunnel syndrome, spinal canal stenosis, spondylosis destruction, fractures, and osteoarthritis. As a treatment for knee osteoarthritis, intra-articular injections of hyaluronic acid and steroids are performed. In general, steroid injections have a strong short-term anti-inflammatory effect, but there is a risk of complications, such as infection. In addition to aging, dialysis patients are prone to weakened immune systems and susceptibility to infection. Therefore, more attention should be paid to the treatment of osteoarthritis in dialysis patients. This study aimed to compare the effects of steroid and complication of infection of dialysis and non-dialysis patients who underwent intra-articular steroid injection.

**Methods:**

A total of 20 dialysis patients (23 knees) and 20 non-dialysis patients (24 knees) with knee osteoarthritis who underwent steroid injections were investigated. All patients underwent radiographic diagnosis and were evaluated for the Western Ontario and McMaster Universities Osteoarthritis Index (WOMAC) score, visual analog scale (VAS), range of motion (ROM), and side effects before, and at 3, and 6 months after injection.

**Results:**

The mean body mass index (BMI) was 21.3 (±standard deviation (SD) 2.8) and 24.9 (±SD 2.6) in dialysis and non-dialysis patients, respectively, showing a significant difference. Both dialysis and non-dialysis patients showed statistically significant improvement in VAS and WOMAC scores after steroid injection. There were no significant differences between dialysis and non-dialysis patients in the gender differences and mean age. There were no infection complications in both groups.

**Conclusions:**

This study revealed the analgesic effect of steroids on knee osteoarthritis in dialysis and non-dialysis patients. On the other hand, there were no infection complications in either patient. These findings suggest that intra-articular steroid injection is safe for dialysis patients.

## Introduction

As of December 31, 2020, the number of hemodialysis patients in Japan is 347,671, the third largest in the world after the United States and China. At the end of 2017, the number of dialysis patients per million people was 2,754.3, the highest in the world, and the average age of hemodialysis patients reached 69.40. The causes leading to dialysis are diabetic nephropathy, chronic glomerulonephritis, and nephrosclerosis.

Hemodialysis patients have various complications, and orthopedic diseases include carpal tunnel syndrome due to dialysis amyloidosis, spinal canal stenosis, spondylosis destruction, fractures, and osteoarthritis ^(1), (2)^. On the other hand, the number of patients with knee osteoarthritis in the world exceeds 300 million, and it is estimated that this number will increase yearly as society ages ^(3)^.

As a treatment for knee osteoarthritis, exercise therapy, such as weight loss, and quadriceps strengthening, is effective in the early stages. If the pain worsens, drug therapy, including non-steroidal anti-inflammatory drugs and acetaminophen, as well as intra-articular injections of hyaluronic acid and steroids, is performed ^(4), (5), (6)^. In general, steroid injections have a strong short-term anti-inflammatory effect, but there is a risk of complications, such as infection, and steroid-induced cartilage atrophy ^(7), (8), (9), (10), (11), (12)^. If conservative treatment is ineffective, surgical treatment, such as high tibial osteotomy or total knee arthroplasty, is considered ^(13)^.

In addition to aging, dialysis patients are prone to weakened immune systems and susceptibility to infection due to various factors, such as the effects of uremic toxins accumulated in the body, nutritional deficiencies, anemia, and diabetes complications. It has been reported that artificial joint surgery in dialysis patients has a high mortality rate ^(14)^, and more attention should be paid to the treatment of osteoarthritis in dialysis patients. To the best of our knowledge, no report has investigated the effects of intra-articular steroid injections on dialysis patients till date.

This study aimed to compare the effects of steroid and complication of infection of dialysis and non-dialysis patients who underwent intra-articular steroid injection.

## Materials and Methods

This prospective study was conducted in the Department of Orthopedic Surgery in the Mitsuhashi Hospital. A total of 20 dialysis patients (23 knees) and 20 non-dialysis patients (24 knees) with knee osteoarthritis who underwent steroid injections were investigated between April 1, 2020, and March 31, 2022. All patients who received steroid injections during the study period were included. Indications for injection are patients with severe knee pain that make life difficult and patients without severe diabetes and suppurative knee arthritis. All patients underwent radiographic diagnosis and were assessed using the Kellgren-Lawrence (KL) classification before and at 3 and 6 months after injection. Povidone-iodine was used for disinfection. All cases received only one intra-articular knee injection of triamcinolone acetonide 20 mg. Injected patients were evaluated for the Western Ontario and McMaster Universities Osteoarthritis Index (WOMAC) score, visual analog scale (VAS), range of motion (ROM), and side effects before, and at 3, and 6 months after injection. Knee ROM was measured with a goniometer in the supine position. The improvement rate of VAS was calculated as the degree of improvement in post-injection pain relative to pre-injection pain. The values are presented as mean ± SD. An independent t-test was performed to compare each item in both groups. A p-value of 0.05 or less was considered statistically significant.

The institutional review board (IRB) of Mitsuhashi Hospital approved the study, and the approval code issued by the IRB was 222110001.

## Results

Among the dialysis patients, 65% were females, while 50% of the non-dialysis patients were females. The mean age was 71.4 (±SD 10.0) and 70.5 (±SD 13.7) years in dialysis and non-dialysis patients, respectively. The dialysis history of dialysis patients was 16.2 (±SD 11.7) years. The mean body mass index (BMI) (kg/m^2^) was 21.3 (±SD 2.8) and 24.9 (±SD 2.6) in dialysis and non-dialysis patients, respectively, showing a significant difference (p < 0.001) (Table 1). The KL classification of the dialysis patient group was as follows: grade 2, 5 knees; grade 3, 14 knees; and grade 4, 4 knees. On the other hand, the KL classification of the non-dialysis patient group was as follows: grade 2, 7 knees; grade 3, 12 knees; and grade 4, 5 knees.

**Table 1.** Gender, Age, and BMI Data of Dialysis and Non-Dialysis Patients (Two-Tailed Student’s t-test).

	Hemodialysis patients (23 knees)	Non-hemodialysis patients (24 knees)	P values
Male/female	7/13	10/10	0.52
Age	71.4 (±SD 10.0)	70.5 (±SD 13.7)	0.78
BMI	21.3 (±SD 2.8)	24.9 (±SD 2.6)	<0.001

Pre-injection ROM (degrees) in dialysis patients was −9.4° (±SD 6.6°) in extension and 110.1° (±SD 11.4°) in flexion, 3 months after injection was −8.7° (±SD 6.8°) in extension and 110.7° (±SD 11.4°) in flexion, and 6 months after injection was −8.8° (±SD 6.8°) in extension and 112.2° (±SD 11.4°) in flexion. On the other hand, pre-injection ROM in non-dialysis patients was −7.3° (±SD 5.5°) in extension and 112.2° (±SD 9.6°) in flexion, 3 months after injection was −6.5° (±SD 5.5°) in extension and 114.0° (±SD 9.2°) in flexion, and 6 months after injection was −6.5° (±SD 5.4°) in extension and 115.0° (±SD 9.2°) in flexion (Table 2 and 3). The VAS improvement rate in dialysis patients was 54.8% (±SD 20.0%) 3 months after injection (p < 0.001) and 51.7% (±SD 17.7%) 6 months after injection (p < 0.001). On the other hand, the VAS improvement rate in non-dialysis patients was 53.3% (±SD 19.0%) 3 months after injection (p < 0.001) and 46.3% (±SD 19.7%) 6 months after injection (p < 0.001) (Table 4). The WOMAC score of dialysis patients was 59.3 (±SD 18.0) before injection, 48.3 (±SD 19.9) 3 months after injection, and 47.5 (±SD 20.2) 6 months after injection (p < 0.05). On the other hand, the WOMAC score of non-dialysis patients was 55.5 (±SD 18.0) before injection, 40.8 (±SD 17.6) 3 months after injection (p < 0.01), and 38.4 (±SD 17.0) 6 months after injection (p < 0.01) (Table 5). As a result, intra-articular injection of corticosteroids was significantly effective in both dialysis and non-dialysis patients.

**Table 2.** Knee Extension Data for Dialysis and Non-Dialysis Patients (Two-Tailed Student’s t-test).

	Extension (Pre-injection)	Extension (Post-injection 3M)	P values	Extension (Post-injection 6M)	P values
Hemodialysis patients	−9.4°(±SD 6.6°)	−8.7°(±SD 6.8°)	0.73	−8.8°(±SD 6.8°)	0.76
Non-hemodialysis patients	−7.3°(±SD 5.5°)	−6.5°(±SD 5.5°)	0.66	−6.5°(±SD 5.4°)	0.66

**Table 3.** Knee Flexion Data for Dialysis and Non-Dialysis Patients (Two-Tailed Student’s t-test).

	Flexion (Pre-injection)	Flexion (Post-injection 3M)	P values	Flexion (Post-injection 6M)	P values
Hemodialysis patients	110.1°(±SD 11.4°)	110.7°(±SD 11.4°)	0.85	112.2°(±SD 11.4°)	0.54
Non-hemodialysis patients	112.2°(±SD 9.6°)	114.0°(±SD 9.2°)	0.50	115.0°(±SD 9.2°)	0.32

**Table 4.** VAS Improvement Rate in Dialysis and Non-Dialysis Patients (Two-Tailed Student’s t-test).

	VAS improvement rate (3M)	P values	VAS improvement rate (6M)	P values
Hemodialysis patients	54.8% (±SD 20.0%)	<0.001	51.7% (±SD 17.7%)	<0.001
Non-hemodialysis patients	53.3% (±SD 19.0%)	<0.001	46.3% (±SD 19.7%)	<0.001

**Table 5.** WOMAC Score Data in Dialysis and Non-Dialysis Patients (Two-Tailed Student’s t-test).

	WOMAC score (Pre-injection)	WOMAC score (Post-injection 3M)	P values	WOMAC score (Post-injection 6M)	P values
Hemodialysis patients	59.3 (±SD 18.0)	48.3 (±SD 19.9)	0.054	47.5 (±SD 20.2)	0.042
Non-hemodialysis patients	55.5 (±SD 18.0)	40.8 (±SD 17.6)	0.006	38.4 (±SD 17.0)	0.001

There were no infection complications in both groups, although the follow-up period was as short as 6 months.

## Discussion

In this study, we found that intra-articular injection of steroid is effective for knee osteoarthritis in dialysis and non-dialysis patients and can be performed relatively safely even in dialysis patients generally susceptible to infection.

Intra-articular injections of steroids have long been used to treat knee pain. As for the mechanism of action, it is believed that knee pain is improved by the action of steroids to calm inflammation in the knee joint. Intra-articular steroid injections generally cause septic arthritis and cartilage damage as complications ^(7), (15), (16)^. To date, it has been reported that the probability of developing septic arthritis following intra-articular injection ranges from 0.037% to 0.0002% ^(17), (18), (19), (20)^. On the other hand, the infection rate in dialysis patients is unclear. Meermans et al. studied 175 patients who underwent total hip arthroplasty (THA) after steroid injection of patients and reported that the risk of infection was not particularly increased ^(21)^. Moreover, Wang et al. analyzed patients who received intra-articular steroid injections before THA or total knee arthroplasty surgery by a meta-analysis and reported that there was no difference in the infection rate compared to the control group ^(22)^. It has also been suggested that intra-articular steroid injections may reduce cartilage degeneration in osteoarthritis patients in the short term ^(23)^.

As mentioned above, dialysis patients often show weakened immunity and susceptibility to infections. First, pathogenic microorganisms are likely to enter the body through angioaccess (internal shunt, indwelling catheter for emergency dialysis) in hemodialysis and peritoneal access (indwelling catheter in the peritoneal cavity) in peritoneal dialysis. In the skin, sweating disorder and epithelial accumulation are observed, and in the respiratory tract, dryness of the mucous membrane and decreased ciliary motility are observed. In the urinary tract, a decrease in the self-cleaning action of the urinary tract due to a decrease in urine output is observed, and there is a so-called disturbance of the skin-mucosal barrier in biological defense ^(24), (25)^.

In chronic renal failure/dialysis patients, neutrophils, monocytes/macrophages, T lymphocytes, and B lymphocytes are abnormal, and such patients are immunocompromised and susceptible hosts ^(26), (27)^.

Therefore, there was concern about the risk of infection with intra-articular steroid injections in dialysis patients susceptible to infection. However, in the results of this study, there was no clear significant difference between dialysis and non-dialysis patients in terms of efficacy and side effects of intra-articular injection of steroids. These findings suggest that intra-articular steroid injection is safe for dialysis patients.

In general, dialysis patients tend to have a lower BMI the longer they have been on dialysis ^(28)^. BMI was lower in dialysis patients, which is believed to be due to anorexia due to uremia and malnutrition due to protein/energy disorders.

This study has some limitations. Compared to the number of samples required, the number of cases this time is too small. Therefore, it is necessary to increase the number of cases and reconsider in the future. Moreover, it may be necessary to consider the number of corticosteroids. In addition, due to the short-term follow-up of 6 months, it is necessary to consider long-term follow-up in the future.

### Conclusions

This study revealed the analgesic effect of steroids on knee osteoarthritis in dialysis and non-dialysis patients. On the other hand, there were no infection complications in either patient.

Although the results of this study demonstrated that intra-articular knee injections for dialysis patients are safe, dialysis patients are susceptible to infections. Therefore, careful disinfection, and procedures are required.

## Article Information

### Conflicts of Interest

None

### Author Contributions

Yusuke Tabata conceived of this study and performed all injections. Yusuke Tabata analyzed all data and performed statistical processing. Yusuke Tabata wrote this manuscript. Satoshi Omori, Osamu Mitsuhashi, Kazuo Enomoto, Yuki Sawano, Noriyoshi Murotani, Koichiro Omori, and Yoichiro Tabata provided useful advice for this manuscript. Tokifumi Majima was a major contributor in writing the manuscript. All authors read and approved the final manuscript.

### Approval by Institutional Review Board (IRB)

All procedures were in accordance with the ethical standards of our hospital and with the 1964 Helsinki Declaration and its later amendments or comparable ethical standards. The institutional review board of Mitsuhashi Hospital approved the study. Approval code issued by the institutional review board (IRB) is 222110001.

### Informed Consent

Informed consent was obtained from all individual participants included in the study.

### Consent to Publish

Consent for publication was obtained from all individual participants included in the study.
